# *Synechocystis*: Not Just a Plug-Bug for CO_2_, but a Green *E. coli*

**DOI:** 10.3389/fbioe.2014.00036

**Published:** 2014-09-18

**Authors:** Filipe Branco dos Santos, Wei Du, Klaas J. Hellingwerf

**Affiliations:** ^1^Molecular Microbial Physiology Group, Faculty of Life Sciences, Swammerdam Institute of Life Sciences, University of Amsterdam, Amsterdam, Netherlands; ^2^Photanol B.V., Amsterdam, Netherlands

**Keywords:** *Synechocystis*, photosynthesis, systems biology, sustainability, genetic engineering

## Abstract

Following multiple reports warning for threats posed by raising levels of atmospheric CO_2_, it is of paramount importance that human society rapidly evolves to be sustainable. Processes relying on photosynthetic microorganisms, converting CO_2_ and water into compounds of interest, fueled by light, are very pertinent, particularly if not directly competing for arable land. Here, we identify specific research questions that remain to be targeted to exploit the full potential of cyanobacterial cell factories. We argue that this approach will be more likely to be successful if organisms such as *Synechocystis* are not perceived as mere chassis for CO_2_ fixation, but rather considered as the “green” *E. coli*.

## Introduction

Atmospheric levels of CO_2_ have steadily increased since the late nineteenth century accelerating to an unprecedented rate from the mid-twentieth century onward. Multiple experts have warned for the consequences posed by the latter. Their tone of urgency has been increasing as early predictions materialize. Signs of consensus seem to be emerging among decision makers, elevating the relevance of environmental sustainability on the political agenda (IPCC, 2014).

Sustainability requires maintenance of chemical balances of human activities, harmonizing them with the existing natural processes (Dhillon and von Wuehlisch, 2013). Human societies are mostly fueled by the oxidation of carbon-containing fossilized deposits, leading to CO_2_ release. One of the efforts to reduce our dependency on fossil fuels focused on exploiting microorganisms to transform plant-derived sugars into bulk chemicals and/or biofuel. Although important toward balancing CO_2_ fluxes, this approach presents several drawbacks. To name but a few, (i) it requires large fractions of arable land, which exerts further pressure on natural resources; (ii) it competes with human food supply; (iii) it leads to a huge amount of biowaste, implying sunlight being used inefficiently. Considerable research efforts have been placed on minimizing these shortcomings. However, advancements still fall short of what is necessary for fully sustainable economies with the current living standards.

The usage of oxygenic photosynthetic prokaryotes as cell factories to directly convert CO_2_ and water into compounds of interest fueled by light and having oxygen as the by-product, presents itself as a very promising alternative. This approach would potentially resolve most of the issues highlighted above, reducing substantially the need of soil and water and leading to less waste. Here, while emphasizing the inherent advantages of this approach, we will expose the areas that require improvement in order to make this a truly economically feasible alternative to current production methods. Focus will be placed on the scientific and technological issues that need further attention to transform lab-scale “proof-of-concept” experiments into full-blown industrial processes.

## Cyanobacterial Photosynthetic Machinery

If one wants to engineer a phototrophic organism into a catalyst for the reaction:

$$CO_{2}+H_{2}O\to \mathrm{product}\left(e\mathrm{.g}\mathrm{.,}C_{2}H_{6}O\right)+O_{2}$$

thereby bypassing biomass formation, one has the choice of using plants (green) algae or cyanobacteria. As genetic accessibility and photosynthetic efficiency are highest in the latter, they are the organisms of choice. This particularly holds for the production of small compounds (e.g., ethanol, butanol) that accumulate extracellularly, not requiring intracellular storage capacity. The situation may be different, for instance, in the production of pharmaceutical proteins, for which green algae may be more profitable because of their larger cytoplasm (Wijffels et al., 2013).

Ultimately, all sustainable processes in which CO_2_ is fixed via oxygenic photosynthesis rely on its efficiency and capacity. As there are differences in this process between pro- and eukaryotic microorganisms, we will discuss those here, pointing out some key advantages of the cyanobacterial photosynthetic machinery, along with key related aspects that remain to be fully elucidated.

### Fundamental advantages

Of the three clades of oxyphototrophs, cyanobacteria have the highest photosynthetic efficiency. It has been shown that in competition experiments between a cyanobacterium and algae, cyanobacteria out-compete the others (Mur et al., 1977). Additionally, where the highest energetic efficiency of C3 and C4 plants has been estimated to be 4.5 and 6% of the incoming solar power, respectively, cyanobacteria can perform significantly better (Janssen et al., 2000). Indirectly, the same conclusion can be drawn from measuring the light intensity at which phototropic microorganisms transit from oxygen consumption to oxygen production (compensation point), also because of their lower maintenance requirements.

Cyanobacteria (along with red algae) contain phycobilisomes, making them more robust in the efficient absorption of polychromatic visible and far-red irradiation. These antennae are suitable targets to engineer increased photosynthetic performance. This can be directed not only at the size, composition, and color of these phycobilisomes (important during scale-up; see further below) but also at the role of the orange carotenoid protein (OCP). The latter is the key component in a mechanism that causes exciton dissipation directly into heat, when cyanobacteria are subjected to over-saturating light intensities.

This OCP-mediated quenching mechanism is one of many regulatory mechanisms that modulate photosynthetic efficiency in oxyphototrophs [see Cardol et al. (2011), Hellingwerf et al. (1994)]. The list includes the zeaxanthin cycle, PSI trimerization, various forms of cyclic electron transfer (see further below), state transitions, circadian regulation of photosystem expression, and chromatic adaptation. However, recently, it has been emphasized that each oxyphototroph only makes use of a limited number of these. For instance, the PS-II repair mechanism in which damaged D1 polypeptides are replaced, rather than replacing the entire photosystem, is absent in *Synechocystis*. Instead, this organism protects itself via a set of flavodiiron proteins that divert electrons from the linear Z-scheme into a cyclic pathway, thereby preventing over-reduction of the cells, when either too much light or too little CO_2_ is available (Bersanini et al., 2014).

The presence of an extensive set of $CO_{2}{\mathrm{∕HCO}}_{3}^{2-}$ uptake systems in cyanobacteria, assures that their oxygenic photosynthesis is not plagued by “photorespiration,” the dissipative process in which the rubisco enzyme mistakes an oxygen molecule for CO_2_. This is another important factor that contributes to their high efficiency of photosynthesis. The small remaining rate of “photorespiration” may have a key function in amino acid biosynthesis in the organism (Bauwe et al., 2012).

### Key questions that remain to be targeted

Unlike cyanobacteria, algae are compartmentalized. This has a significant impact on the tools available for synthetic biology, with clear advantages for cyanobacteria. Nevertheless, each compartment catalyzes a characteristic part of the metabolism of eukaryotes, each with its specific regulation. Although often a disadvantage, this can sometimes be exploited. Engineering fatty acid biosynthesis for increased biodiesel production (Weselake et al., 2008) is an example of this successful exploitation of a compartmentalized cell.

**Figure 1:**
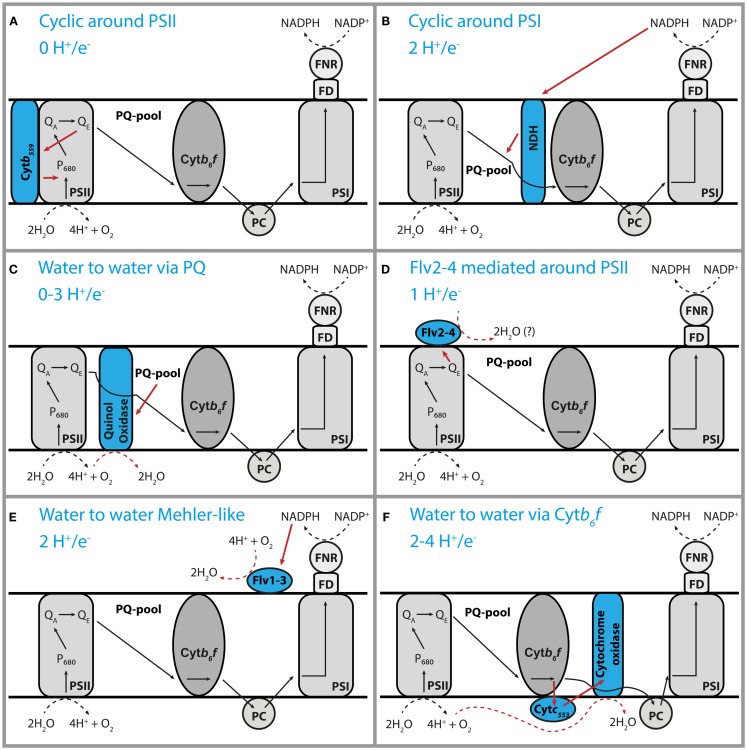
**Inventory of pathways for cyclic electron flow in cyanobacteria**. Schematic representation of six different forms of cyclic electron transfer that may occur in cyanobacteria **(A–F)**. The tentative H^+^/e^−^ stoichiometry of each form of cyclic e^−^ transfer is indicated. The electron acceptor of the Flv2–4 protein has not been characterized yet. This cyclic transfer of electrons may make the NADPH and ATP output of the photosynthesis machinery different from the 3/2 textbook ratio.

The redox state of the plastoquinone pool in the thylakoid membranes has been implied as the regulatory parameter in many key processes in oxyphototrophs (Allen, 2005). In chloroplasts, the balance of electron transfer from PS-II to PSI straightforwardly determines this redox state. In cyanobacteria, there are additional pathways that are important (Figure 1), for instance, the supply of electrons by the main dehydrogenase (NDH-1) and withdrawal by the respiratory oxidases (Schuurmans et al., 2014). Consequently, it is much more complex to understand the control of the PQH_2_/PQ redox state in cyanobacteria.

This redox state has often been derived from pulse-amplitude modulated fluorescence (PAM) measurements. We have recently reported that PAM is fraught with artifacts when applied to *Synechocystis* (Schuurmans et al., 2014), and that the redox state is strictly controlled around its midpoint potential (except when cells are leaving lag-phase). The PAM signals most likely reflect the redox state of the primary quinone of PS-II (Q_A_). Among the artifacts is a significant contribution of phycobilisome fluorescence to the PAM signals (Schuurmans et al., 2014). Proper interpretation of these PAM signals is important because many aspects of photosynthesis can be derived from them.

One characteristic that is derived from PAM measurements is the “rate of cyclic electron flow” (Fan et al., 2007), i.e., the rate at which electrons follow a cyclic path around PSI via an electron transfer reaction between NDH-1 and plastoquinone. While consuming part of the light energy, this will lead to production of ATP only (i.e., without coupled NADPH formation), and therefore, alter the ratio of synthesis of these high-free-energy intermediates (Hellingwerf and Konings, 1985). In some situations, as under salt stress, the cell uses this process to its advantage. They initiate high rates of cyclic electron flow to generate extra ATP to drive transport reactions (van Thor et al., 2000). Moreover, cells are much more complex than depicted in many textbook representations, as multiple forms of cyclic electron transfer can occur, with rates that are strongly dependent on the physiological state of the cell (Figure 1). As each of these has its own specific proton:photon stoichiometry, it is nearly impossible to estimate the ratio of production of NADPH and ATP *in vivo*. It will be very important for a better understanding of the energetics and metabolism of cyanobacteria to find ways to measure this ratio.

Next to NADPH, also NADH is an important reduced cofactor in cyanobacterial cells. The redox potential of the latter is often reported to be higher than of the NADPH/NADP^+^ couple, although few solid data are available on this. Many (engineered) metabolic pathways require reducing equivalents. In cyanobacteria, the preferred cofactor would be NADPH, considering the driving force of the reaction, but many high-capacity (catabolic) reductases are NADH-specific. Redox cofactor matching is therefore important in design of product formation by cyanobacteria.

## Synthetic Biology Toolbox

### Requirements specific to photosynthetic organisms

*Synechocystis* is a great chassis to engineer the production of compounds directly from CO_2_. This requires modification of its metabolic network, by removal and/or addition of components via genetic modification. This network contains many anabolic pathways, which ensures that the carbon fixed is efficiently channeled into biomass. Engineered pathways tap at convenient hub metabolites, often with detrimental effects on growth (Nogales et al., 2012; Savakis et al., 2013). For this reason, it is crucial to design synthetic circuits that are stabilized by expressing the necessary enzymatic machinery and/or regulatory switches at just the right moment. Considering the available technology for large-scale cultivation, the improvement of induction systems responsive at high-cell densities would be most useful, reducing tension between anabolic and product formation pathways.

### State-of-the-art

Some cyanobacteria are naturally transformable, making them very attractive photosynthetic production hosts. Contrary, their polyploidy (Griese et al., 2011) requires additional efforts to ensure full segregation after mutagenesis and presents additional challenges during the generation of gene disruption libraries (Tyo et al., 2009). Here, we will focus on the state-of-the-art methods to genetically modify these organisms and regulate their gene expression.

#### Genetic modifications

Markerless gene knock-out/in strategies for cyanobacteria are attractive because of the limited number of resistance cassettes available. Several methods have been successfully applied (Table 1). Among these, the *sacB*-sucrose method is widely used, but its application is limited to glucose-tolerant strains, while the *mazF*-nickel method can circumvent this. The *acsA*-acrylate method was developed for *Synechococcus* PCC7002, but may also be useful for other cyanobacteria. However, it has the drawback of possibly generating undesired mutations. The *upp*-5-fluorouracil method works according to a similar principle, such that *upp* or *acsA* need to be inactivated first in order to make cells sensitive to 5-fluorouracil or insensitive to acrylate, respectively. The *upp*-5-fluorouracil method is considered more efficient because 5-fluorouracil can inhibit cells that contain even a single copy of *upp*. In addition to these counter-selection methods, the Flp–FRT (recombinase and recognition site, respectively) based method also works, provided the Flp plasmid can be lost quickly. Another strategy consists of using essential-cyanobacterial gene complementation for plasmid maintenance, like *recA* (Akiyama et al., 2011), but this strategy is limited by plasmid maintenance. Recent reports characterize cyanobacterial CRISPR–Cas systems (Hein et al., 2013; Kopfmann and Hess, 2013; Scholz et al., 2013). Probably, mutants obtained using the latter will be reported soon. Use of TALEN systems can be expected soon after (Montague et al., 2014).

**Table 1:** **Markerless gene knock-out and knock-in methods for cyanobacteria**.

Methods	Strains tested	Reference
*sacB*-sucrose	*Synechocystis* sp. PCC6803 (only glucose-tolerant strain)	Lea-Smith et al. (2014), Viola et al. (2014)
*mazF*-nickel	*Synechocystis* sp. PCC6803	Cheah et al. (2012)
*acsA*-acrylate	*Synechococcus* sp. PCC7002	Begemann et al. (2013)
*upp*-5-Fluorouracil	*Synechococcus elongatus* PCC7942	Aikens and Turner (2013), Xu and Green (2012)
	*Synechocystis* sp. PCC6803	
	*Synechococcus* sp. PCC7002	
Flp–FRT	*Synechococcus elongatus* PCC7942	Tan et al. (2013)
	*Synechocystis* sp. PCC6803	

#### Regulation of gene expression

Expression can be regulated either through inducible promoters or other inducible elements, such as riboswitches. Riboregulators may include two parts: a cis-repressed mRNA, which forms a stem–loop structure that occludes the ribosomal binding site and prevents target gene translation, and a trans-activating RNA. A set of riboswitches has been developed for *Synechocystis*, which achieves 13-fold expression differences (Abe et al., 2014).

A large number of systems inducible by metal ions, metabolites, and macronutrients have already been characterized (Berla et al., 2013). One remaining challenge is that inducers, because of leakiness or limited dynamic range, cannot sufficiently control expression. Research on the inducing mechanism might present a possible solution to these problems. For example, through a single mutation in the core domain of the *lac* repressor in *E. coli* (Gatti-Lafranconi et al., 2013) or the use of a DNA-looping promoter in *Synechocystis* (Camsund et al., 2014), a wider dynamic range and decreased leakiness was reported for LacI-repressed promoters. Alternatively, with degradation tags, mRNA, and hence protein-lifetime can be modulated (Huang et al., 2010).

## Mathematical Modeling

### Lessons learnt from metabolic engineering of fermentative organisms

Systems biology approaches, i.e., the iterative combination of experimental data with theoretical modeling aiming at a systems level understanding, have been gaining pertinence in metabolic engineering (Teusink and Smid, 2006). The importance of computational biology extends beyond helping us deal with large-data sets. Although simplistically portrayed as such, pathways engineered for the production of compounds of interest are never “plug‘n’play.” They always have repercussions to the host (Branco dos Santos et al., 2013), which therefore should not be perceived as “plug-bugs.” This is an important message from the first biotechnological processes for fermentative organisms – engineering cyanobacteria is more likely to succeed if the whole host and its interactions with the environment and neighboring cells are considered.

### Making (useful) predictions of phenotypic responses

Models useful for cyanobacteria are very diverse and should be chosen depending on their purpose. Whether mathematical descriptions should contain mechanistic detail or remain phenomenological depends on the data available. It is important to consider whether a model will be probed to predict the outcome of perturbations to a given state, or rather to simulate steady-state conditions. The boundaries of any model should be stipulated, accepting that all models are only valid under certain (preferably explicit) assumptions.

#### Mechanistic vs. phenomenological

As illustrated above, certain aspects of the photosynthetic machinery of cyanobacteria still remain elusive. The dynamics of this central process will undoubtedly affect all other rates in a cell, production rate of target compounds included. Does this mean that efforts to model these organisms are futile? Not in our perspective – with the right data one can approximate energy conversion during photosynthesis, generating useful predictions that help understand other processes for which mechanisms are known. The level of mechanistic detail included in a model should be carefully pondered. Too much leads to parameter uncertainty while too little results in predictions that provide little insight. There is one particular modeling strategy that would be interesting to see applied to cyanobacteria – whole cell economy. Understanding the cost/benefit relationship between having different modes available in different environments can shed light on how and why specific pathways are regulated and what trade-offs cells face (Molenaar et al., 2009).

#### Dynamic vs. steady-state models

Besides the purpose of the model itself, whether or not to include time as an explicit dimension in a model depends mostly on how much kinetic information is available (Santos et al., 2011). Stoichiometric models of cyanobacterial metabolism outnumber kinetic ones. At least three factors are behind this: (i) models of these organisms are often used to simulate “steady-state” fluxes toward biomass and products, given a fixed input of light and nutrients (Nogales et al., 2012); (ii) the kinetics of key reactions remain elusive, most notably cyclic electron transfer; (iii) there is a lack of standardized data sets that would allow accurate estimation of parameters. Nevertheless, to make kinetic models of these organisms is very pertinent, both for basic science and various applications (Young et al., 2011). These allow a better understanding of how cells respond to a perturbation, and carry flux through different routes.

#### Model size matters

Models should not be a complete description of all processes in a cell, but rather a description of those necessary to fulfill its purpose. Most cyanobacteria have extended anabolic versatility compared to fermentative organisms, resulting in few auxotrophies. Expression of genes encoding enzymes involved in anabolic pathways varies depending on external conditions (Mitschke et al., 2011). When simulating growth is the purpose, which usually is for cyanobacteria, it is unsurprising that genome-scale models are most popular (Feist and Palsson, 2010). However, by lumping reactions, insightful smaller models can be created (Kelk et al., 2012). These might eventually allow predicting dynamic behavior with reduced uncertainty.

## From Proof-of-Concept to Industrial Scale

Biotechnological applications with photosynthetic prokaryotes amount to a few M€ annually (http://www.algaeindustrymagazine.com/). Though this is the modest amount if compared to oil-producing eukaryotic algae and hetero-fermentative microorganisms, for reasons already discussed, it is expected (and urgently needed) that this value will gradually increase during the next decade. So, how to make proof-of-principle studies blossom into large-scale sustainable industrial processes?

### Challenges and opportunities during the scale-up

Compared to fermentative organisms, which have been exploited by humans for at least over 8 millennia (Salque et al., 2013), the rational usage of photosynthetic prokaryotes is in its infancy [a notable exception being their food and feed applications (Habib et al., 2008)]. The resulting lack of empirical knowledge hampers their economic attractiveness. Albeit issues such as preventing contaminations and improving downstream processing can be improved, arguably, the major limitation remains light supply. This issue is being addressed by attempting to improve light penetration into photobioreactors, e.g., through antenna-size reduction (Kirst et al., 2014; Lea-Smith et al., 2014). This can also be achieved by higher carbon partitioning toward product rather than biomass, if clever production hosts are engineered (Liu et al., 2011).

## Final Remarks

The development of truly sustainable biotechnological processes relying on photosynthetic prokaryotes is a promising solution against the major environmental challenge faced by humanity. Increased understanding of the physiology of these organisms has the highest priority. This can be rapidly achieved if research approaches successfully used in heterotrophic microorganisms are increasingly applied to prokaryotic oxyphototrophs. This will be more successful if organisms such as *Synechocystis* are not perceived as mere chassis for CO_2_ fixation, but rather treated as “green” *E. coli*. The input of many research efforts will allow for a much more complete understanding, ultimately, resulting in the development of improved sustainable cell factories.

## Conflict of Interest Statement

Klaas J. Hellingwerf is the scientific advisor of Photanol B.V., a University of Amsterdam spin-off company aiming at commercializing sustainable applications with cyanobacteria. The other co-authors declare that the research was conducted in the absence of any commercial or financial relationships that could be construed as a potential conflict of interest.
