# Corrigendum: *Synechocystis*: not just a plug-bug for CO_2_, but a green *E. coli*

**DOI:** 10.3389/fbioe.2016.00032

**Published:** 2016-04-13

**Authors:** Filipe Branco dos Santos, Wei Du, Klaas J. Hellingwerf

**Affiliations:** ^1^Molecular Microbial Physiology, Faculty of Life Sciences, Swammerdam Institute of Life Sciences, University of Amsterdam, Amsterdam, Netherlands; ^2^Photanol B.V., Amsterdam, Netherlands

**Keywords:** *Synechocystis*, photosynthesis, systems biology, sustainability, genetic engineering

In our publication (Branco Dos Santos et al., 2014), a statement may lead to the wrong conclusion that PSII repair mechanisms are entirely absent in *Synechocystis* (page 2, paragraph 3). The authors here would like to clarify that they merely wanted to suggest that under the conditions most often used in physiological studies on the organism in the lab, alternative ways to deal with light inhibition (and/or CO_2_ limitation) are mostly used. There is a large body of evidence supporting that PSII repair mechanisms indeed are present in *Synechocystis* and that they do play an active functional role, particularly under high incident light intensities (Silva, 2003; Komenda et al., 2006; Sacharz et al., 2015), which we did not mean in anyway to ignore or undervalue. In line with the original message of the paper, indeed, this is another factor that should be considered when designing optimal green cell factories.

## Author Contributions

FBS, WD, and KH were involved in preparing the correction.

## Conflict of Interest Statement

KH is the scientific advisor of Photanol B.V., a University of Amsterdam spin-off company aiming at commercializing sustainable applications with cyanobacteria. FBS and WD have no conflict of interest to declare.
